# SLFN11 Restricts LINE-1 Mobility

**DOI:** 10.3390/cells14110790

**Published:** 2025-05-28

**Authors:** Zhongjie Ye, Yuqing Duan, Ao Zhang, Zixiong Zhang, Saisai Guo, Qian Liu, Dongrong Yi, Xinlu Wang, Jianyuan Zhao, Quanjie Li, Ling Ma, Jiwei Ding, Shan Cen, Xiaoyu Li

**Affiliations:** 1 Institute of Medicinal Biotechnology, Chinese Academy of Medical Sciences and Peking Union Medical College, Beijing 100050, China; zj.ye1@siat.ac.cn (Z.Y.); s2022010034@student.pumc.edu.cn (Y.D.); za1632649341@163.com (A.Z.); zixiongzhang@outlook.com (Z.Z.); guosaisai@imb.pumc.edu.cn (S.G.); j2024080001@pumc.edu.cn (Q.L.); dongrong.yi@imb.pumc.edu.cn (D.Y.); zhaojianyuan@imb.pumc.edu.cn (J.Z.); quanjie.li@imb.pumc.edu.cn (Q.L.); maling26@imb.pumc.edu.cn (L.M.); shancen@imb.pumc.edu.cn (S.C.); 2 Shenzhen Institute of Advanced Technology, Chinese Academy of Sciences, Shenzhen 518055, China; 3 Key Laboratory of Biomacromolecules (CAS), CAS Center for Excellence in Biomacromolecules, Institute of Biophysics, Chinese Academy of Sciences, Beijing 100101, China; wang_xl1978@aliyun.com

**Keywords:** long interspersed element-1, LINE-1, transposon, SLFN11, helicase, RNA polymerase II, heterochromatin, epigenetics

## Abstract

Long interspersed element-1 (LINE-1) is the only active autonomous transposon comprising about 17% of human genomes. LINE-1 transposition can cause the mutation and rearrangement of the host’s genomic DNA. The host has, therefore, developed multiple mechanisms to restrict LINE-1 mobility. Here, we report that SLFN11, a member of the Schlafen family, can restrict LINE-1 retrotransposition, and the inhibitory activity requires its helicase domain. Mechanistically, SLFN11 specifically binds to the LINE-1 5′ untranslated region (5′UTR) and blocks RNA polymerase II recruitment, thereby suppressing its transcription. Furthermore, SLFN11 promotes heterochromatinization, suggesting an epigenetic inhibition pathway.

## 1. Introduction

LINE-1 is the only currently active autonomous transposon in the human genome. Approximately 500,000 LINE-1 copies occupy about approximate 17% of the human genome, with about 100 retrotransposition-competent elements remaining active [1]. The retrotransposition of full-length LINE-1 elements is initiated with transcription mediated by the internal RNA polymerase II promoter located in the 5′UTR region. LINE-1 contains two open reading frames (ORF1 and ORF2) [2]. ORF1 encodes a nucleic acid chaperone protein [3], whereas ORF2 encodes a 150 kDa protein exhibiting both endonuclease (EN) and reverse transcriptase (RT) activities, which are essential for LINE-1 retrotransposition [4]. Both LINE-1-encoded proteins demonstrate cis-preference by binding their cognate RNA to form a ribonucleoprotein (RNP) complex critical for the reverse transcription process. Following nuclear entry, the LINE-1 endonuclease introduces a single-strand nick at a degenerate consensus sequence (5′TTTT/A or variant) to liberate a 3′ hydroxyl residue that serves as a primer for LINE-1 reverse transcriptase to mediate cDNA synthesis and integration through target-primed reverse transcription (TPRT) [2,3].

LINE-1 elements have significantly impacted the host genomes over millions of years by regulating gene expression through various mechanisms, including chromatin structure modification, transcriptional regulation, pre-mRNA processing, and mRNA metabolism [4]. These mobile genetic elements have played a fundamental role in shaping genome architecture and evolution [5,6]. However, ongoing LINE-1 retrotransposition can induce deleterious effects on the host genome. In germline or certain somatic cells, LINE-1 insertion creates insertional mutations, genomic deletions, transduction events, structural rearrangements, and transcriptional interference through premature pausing or termination, potentially disrupting gene function and contributing to various human genetic diseases [7,8]. Furthermore, LINE-1 activity has been implicated in the pathogenesis of certain cancers, autoimmune diseases, and neuropsychiatric diseases [9,10,11].

Host cells, therefore, involve multiple strategies to defend against this endogenous “parasite” [12]. In normal human somatic cells, the transposition of LINE-1 is tightly controlled at every stage of its life cycle through different restriction mechanisms: RNA-induced silencing complexes (RISCs) [13,14], APOBEC3 proteins [15,16,17], TREX1 [18], and Ribonuclease L [19] target LINE-1 nucleic acid metabolism, while MOV10 [20] and SAMHD1 [21] interfere with LINE-1 ribonucleoprotein (RNP) formation and the assembly of stress granules.

Nuclear host factors primarily regulate LINE-1 at the transcriptional level. The human silencing hub (HUSH) complex, for instance, binds to LINE-1 elements and recruits the histone methyltransferase SETDB1 to deposit H3K9me3 marks at LINE-1 loci [22,23,24]. SIRT6, a member of the sirtuin protein family, interacts with the 5′-UTR of LINE-1 and mono-ADP-ribosylates the nuclear co-repressor KAP1 (KRAB-associated protein 1). This modification enhances KAP1 binding to heterochromatin protein HP1α, promoting LINE-1 sequestration into transcriptionally repressive heterochromatin domains [25]. In contrast, cytoplasmic host factors exert post-transcriptional control over LINE-1. For example, MOV10 (Moloney leukemia virus 10 protein) and ZAP (zinc-finger antiviral protein) co-localize with LINE-1 RNPs in cytoplasmic granules and trigger LINE-1 RNA degradation [20,26].

The Schlafen (SLFN) family, initially identified as lymphocyte development regulators [27], comprises 10 murine and 6 human proteins classified into three subgroups by size and domain architecture [28]. Subgroups I-II localize cytoplasmically, while subgroup III members possess nuclear localization signals [29]. Subgroup III SLFNs feature an N-terminal slfn core with AAA-4 nuclease, a central SWAVDL linker, and C-terminal helicase-like domains [29]. Subgroups I-II lack C-terminal regions containing smaller proteins. All human SLFNs share the conserved “slfn box”—a functionally uncharacterized domain homologous to COG2865 in transcription regulators and RNA/DNA helicases. The AAA domain in SLFN5/11/13/14 exhibits ATPase activity-regulating proliferation, differentiation, chromatin dynamics, replication arrest, and apoptosis [30,31]. SLFN11, a subgroup III member with Superfamily 1 helicase homology [28], shows unique anticancer relevance [32]. Besides enhancing cancer cell sensitivity to DNA-damaging agents [33], it modulates cytoplasmic translation and proteotoxic stress [34,35]. Mechanistically, SLFN11 was reported to promote stalled fork degradation, inhibit checkpoint maintenance, disrupt DNA repair checkpoints, and increase chromatin accessibility under replication stress, inducing cell death [32,36].

As an interferon-stimulated gene, SLFN11 inhibits retroviruses (PFV, EIAV, and HIV-1) and flaviviruses (WNV, DENV, and ZIKV) by restricting codon-optimized tRNA availability via tRNA binding/cleavage [33,37,38,39] and impairing ATM/ATR kinase translation [40]. Its ATPase/helicase activities and dephosphorylation are essential for its antiviral function [38]. Since SLFN11 has inhibitory activity against the exogenous retrovirus, it might be interesting to identify whether SLFN11 is also involved in restricting endogenous retroelements, especially LINE-1.

In this study, we investigated the effect of SLFN11 on LINE-1 retrotransposition and showed that SLFN11 inhibits LINE-1 by facilitating heterochromatin formation and blocking the recruitment of RNA polymerase II to LINE-1. This inhibitory activity is closely linked to the helicase activity of SLFN11.

## 2. Materials and Methods

### 2.1. Plasmids and Antibodies

The IAP-neo^TNF^, MusD-neo^TNF^, and CMV-L1-neo^RT+^ reporters and L1-FL DNA were used as previously described [41,42,43]. IAP-FL or MusD-FL was constructed by inserting the 5′-UTR sequence of IAP or MusD upstream of the firefly luciferase reporter gene in the pGL3-Basic vector (Promega, Madison, WI, USA), respectively. Myc-ORF1p, Myc-SLFN11, and Flag-SLFN11 cDNA sequences were cloned into the pcDNA4.0/Zeo(+) vector (Invitrogen, Carlsbad, CA, USA). Site-directed mutagenesis was performed to generate CMV-L1-neoRT− (D702Y mutation in ORF2p), Flag-SLFN11 mutants (K605M, D668A and K605M/D668A), and N-terminal and C-terminal truncation variants (dC 1-332, dN 333-901), which were constructed by standard molecular biology techniques. Commercial antibodies included the following: SLFN11 (sc-374339, Santa Cruz Biotechnology, Dallas, TX, USA; HPA023030, Sigma-Aldrich, St. Louis, MO, USA), H1.2 (ab4086), H3K9me3 (ab8898), H3K27me3 (ab6002), RBBP7 (ab259957), RNA polymerase II (ab5408; all from Abcam, Cambridge, UK); Flag (14793), HA (3724S), Myc (2276), HP1α (2616), EZH2 (5246) (Cell Signaling Technology, Danvers, MA, USA); and Flag (B1020, Biodragon, Jiangsu, China).

### 2.2. Cell Culture and Transfection

Human embryonic kidney 293T cells, 293FT cells, and HeLa cells (ATCC, Manassas, VA, USA) were maintained in DMEM supplemented with 10% fetal bovine serum (FBS; Invitrogen, Carlsbad, CA, USA). Du145 and MCF7 (ATCC) were grown in RPMI-1640 with 10% FBS. Transfections were performed using Lipofectamine 3000 (Invitrogen, Carlsbad, CA, USA) or VigoFect (Vigorous Biotechnology, Beijing, China) according to the manufacturer’s protocols.

### 2.3. LINE-1 Retrotransposition Assay

Cells were seeded in six-well plates 24 h before transfection with 500 ng reporter plasmids (CMV-L1-neo^RT+^, IAP-neo^TNF^, or MusD-neo^TNF^) and indicated the amount of SLFN11, and MOV10 or its empty vector reporter plasmid pcDNA4.0 control. At 48 h post-transfection, cells were trypsinized and reseeded into six-well plates at serial dilutions [43]. The cells were then selected with 0.8 mg/mL G418 (Thermo Fisher, Waltham, MA, USA). After 12 days of selection, colonies were fixed with methanol and stained with 0.5% crystal violet (in 25% methanol). The number of G418-resistant cell colonies was used to quantify retrotransposition events.

### 2.4. Luciferase Assay

Cells were seeded in 6-well plates 24 h before transfection with 500 ng of either L1-FL, IAP-FL, or MusD-FL DNA, along with the indicated amount of SLFN11 or empty reporter vector and 20 ng of pCMV-Renilla [43,44]. Firefly luciferase activity was measured 48 h post-transfection using the Dual-Luciferase Reporter Assay System (Promega, Madison, WI, USA) according to the manufacturer’s instructions. Relative luciferase activity was quantified using the Promega Luciferase Assay Kit (Promega, Madison, WI, USA) on a Berthold microplate reader.

### 2.5. Quantitative RT–PCR

Cells transfected with 500 ng of CMV-L1-neo^RT+^, 500 ng of CMV-L1-neo^RT−^, and 300 ng of SLFN11 or a vector were harvested 48 h post-transfection. Total RNA was extracted using the TRIzol reagent (Invitrogen) and treated with DNase I (RNase-free; Thermo Fisher Scientific, Waltham, MA, USA) to eliminate genomic DNA contamination. Reverse transcription was performed with 1 μg RNA using M-MLV reverse transcriptase (Promega, Madison, WI, USA) and oligo dT primers. qPCR amplification was carried out using the SYBR Green Master Mix (Roche, Basel, Switzerland) on an Applied Biosystems QuantStudio 1 Real-Time PCR System. Primer sequences were as follows:

GAPDH:

Forward: 5′-GGTATCGTGGAAGGACTCATGAC-3′

Reverse: 5′-ATGCCAGTGAGCTTCCCGTTCAG-3′,

LINE-1 mRNA:

Forward: 5′-CTGAAGCGGGAAGGGACTG-3′

Reverse: 5′-CCTTGAGCCTGGCGAACAG-3′,

LINE-1 5′-UTR:

Forward: 5′-CGAGATCAAACTGCAAGGCG-3′

Reverse: 5′-CCGGCCGCTTTGTTTACCTA-3′

LINE-1 ORF1p:

Forward: 5′-ACCTGAAAGTGACGGGGAGA-3′

Reverse: 5′-CCTGCCTTGCTAGATTGGGG-3′ [45].

### 2.6. Western Blotting

Cells were harvested and lysed in an RIPA buffer (150 mM NaCl, 10 mM Tris [pH 7.5], 0.1% SDS, 1% Triton X-100, 1% sodium deoxycholate, 1 mM EDTA) supplemented with protease inhibitors (Roche) for 1 h on ice. Lysates were centrifuged at 12,000 rpm for 10 min at 4 °C, and supernatants were boiled in a 1× loading buffer at 95 °C for 5 min. Equal amounts of cell lysates (10 μg) were separated by SDS-PAGE. Proteins were transferred onto PVDF membranes (Millipore, Boston, MA, USA). Membranes were blocked with 5% non-fat milk in TBST (Tris-buffered saline with 0.1% Tween-20) for 1 h at room temperature, followed by incubation with the primary antibody in 1× TBST overnight at 4 °C, followed by incubation with an appropriate HRP-conjugated secondary antibody (1:5000 dilution; Cell Signaling Technology, Danvers, MA, USA) for 1 h at room temperature. Signals were detected using the SuperSignal West Pico Chemiluminescent Substrate (Thermo Fisher, Waltham, MA, USA).

### 2.7. Co-Immunoprecipitation

Cells transfected with Flag-tagged SLFN11, Myc-tagged LINE-1 ORF1p, or Flag-tagged MOV10 were lysed in an NETN buffer (20 mM Tris-HCl, pH 8.0, 100 mM NaCl, 1 mM EDTA, 0.5% NonidetP-40) containing a protease inhibitor cocktail (Roche, Basel, Switzerland). Lysates were centrifuged at 13,000× *g* for 30 min at 4 °C, and supernatants were treated with RNase A (100 μg/mL; Thermo Fisher Scientific) or left untreated [43]. For immunoprecipitation, 500 μg of lysate was incubated with a 2 μg anti-Flag M2 antibody (Sigma-Aldrich) or control IgG for 4 h at 4 °C, followed by incubation with protein A + G agarose gel beads (Santa Cruz Biotechnology, Dallas, TX, USA) overnight with gentle rotation at 4 °C. Beads were washed five times with the NETN buffer, and the bound proteins were eluted for immunoblotting examination.

### 2.8. Immunofluorescence Microscopy

Cells were co-transfected with Flag-tagged SLFN11 and Myc-tagged LINE-1 ORF1p expression plasmids. Forty-eight hours after transfection, the cells were fixed with 4% paraformaldehyde and permeabilized with 0.1% Triton X-100 in 4% paraformaldehyde for 10 min at room temperature, followed by permeabilization with 0.1% Triton X-100 in PBS for 10 min. After blocking with 5% bovine serum albumin (BSA), the cells were incubated with antibodies against FLAG (mouse monoclonal, 1:500; Sigma-Aldrich, St. Louis, MO, USA) or Myc (rabbit monoclonal, 1:500; Cell Signaling Technology, Danvers, MA, USA) for 2 h at room temperature. The Alexa Fluor 488-conjugated secondary anti-mouse antibody (1:2000 dilution) or Alexa Fluor 594-conjugated secondary anti-rabbit antibody (1:2000 dilution) were used as secondary antibodies for 1 h at room temperature. Nuclei were then stained with DAPI (4′,6-diamidino-2-phenylindole). The images were obtained using the Zeiss Pascal laser scanning confocal microscope (Zeiss, Oberkochen, Germany).

### 2.9. Chromatin Immunoprecipitation

Cells transfected with Flag-tagged SLFN11 were subjected to ChIP assays ChIP using the EZ-CHIP Kit (Cat#17-371, Millipore, Boston, MA, USA) according to the manufacturer’s protocol. Briefly, a minimum of 5 × 10^6^ cells per immunoprecipitation (IP) were crosslinked with 1% formaldehyde, quenched with glycine, and lysed. Chromatin was sonicated to 200–500 bp fragments. Lysates were incubated overnight at 4 °C with antibodies against FLAG (Cat#14793, rabbit monoclonal antibody, Cell Signaling Technology, Danvers, MA, USA), RNA polymerase II (Abcam, ab5408, mouse monoclonal antibody), or the IgG control (Millipore, Boston, MA, USA). Protein–DNA complexes were captured using protein A/G magnetic beads before being washed and eluted. DNA was purified and quantified by qPCR using primers spanning the LINE-1 locus (L1 PCR1–7; sequences below). Input (2% of total chromatin) served as a normalization control. The primer sequences are given below (L1 PCR3 was newly designed; others from Meyer et al.) [25]:

L1 PCR1:

Forward: 5′-AAGATGGCCGAATAGGAACAG-3′

Reverse: 5′-TTTGACTCGGAAAGGGAACTC-3′

L1 PCR2:

Forward: 5′-ACGAGACTATATCCCACACCT-3′

Reverse: 5′-GCAGAGGTTACTGCTGTCTT-3′;

L1 PCR3:

Forward: 5′-ATCTGAGAACGGGCAGACAG-3′

Reverse: 5′-AGCTGCAGGTCTGTTGGAAT-3′;

L1 PCR4:

Forward: 5′-CGATGCGATCAACTGGAAG-3′

Reverse: 5′-GGCCTGCCTTGCTAGATT-3′;

L1 PCR5:

Forward: 5′-CAGAGACACACATAGGCTCAAA-3′

Reverse: 5′-AATCTGGGTGCTCCTGTATTG-3′;

L1 PCR6:

Forward: 5′-ACTCATCTGACAAAGGGCTAAT-3′

Reverse: 5′-CCTATTTCTCCGCATCCTCTC-3′;

L1 PCR7:

Forward: 5′-AATGAGATCACATGGACACAGGAAG-3′

Reverse: 5′-TGTATACATGTGCCATGCTGGTGC-3′.

Relative protein binding on different domains of LINE-1 was calculated after normalization to the input.

### 2.10. Micrococcal Nuclease Assay

Cells transfected with 3 μg of SLFN11 or control plasmids for 60 h were harvested, and nuclei were isolated using the EZ Nucleosomal DNA Prep Kit (Cat#D5220, Zymo Research, Orange County, CA, USA) following the manufacturer’s protocol. In brief, the nuclei were isolated from culture cells and treated with micrococcal nuclease at 42 °C for 20 min. Reactions were terminated with 10 mM EDTA. DNA was purified and analyzed by gel electrophoresis.

### 2.11. shRNA Knockdown Assay

Lentiviruses encoding two independent shRNAs targeting SLFN11 (shRNA6 and shRNA8 are 5′-CCG GCC GAT AAC CTT CAC ACT CAA ACT CGA GTT TGA GTG TGA AGG TTA TCG GTT TTT TG-3′ and 5′CCG GCA GTC TTT GAG AGA GCT TAT TCT CGA GAA TAA GCT CTC TCA AAG ACT GTT TTT TG-3′) or the non-targeting control shRNA were transduced into cells plated in 10 cm dishes. After 36 h, the cells were subject to puromycin selection (the final concentration was 2 μg/mL) for several days until monocolonies were formed in 96-well plates. The cells of monocolonies were examined by Western blot.

### 2.12. IP-MS

Cells co-transfected with CMV-L1-neo^RT+^ and SLFN11 or control plasmids or control plasmids were lysed with a commercialized NP-40 Lysis Buffer (Cat#P0013F, Beyotime Biotechnology, Shanghai, China) on ice for 1 h and then centrifuged at 12,000 rpm for 20 min to collect the supernatant. Cell lysates were incubated with the anti-Flag antibody for immunoprecipitation at 4 °C overnight, and protein A/G agarose beads (Cat#P2197S, Beyotime Biotechnology, Shanghai, China) were added, followed by incubation at 4 °C for 1–3 h. A washing buffer was used to wash the protein–antibody–bead complex multiple times to remove non-specifically bound proteins. The bound proteins were eluted with FLAG peptide (0.1 mg/mL). Elutes were submitted to a commercial service for LC-MS/MS analysis.

### 2.13. Statistical Analysis

Data are presented as means ± standard deviations (SDs). The figures indicate the significance of differences. Statistical analyses were conducted using a two-tailed, unpaired Student’s *t*-test (* *p* < 0.05; ** *p* < 0.01; *** *p* < 0.001; ns, not significant).

## 3. Results

### 3.1. SLFN11 Inhibits LINE-1 Retrotransposition

**Figure 1:**
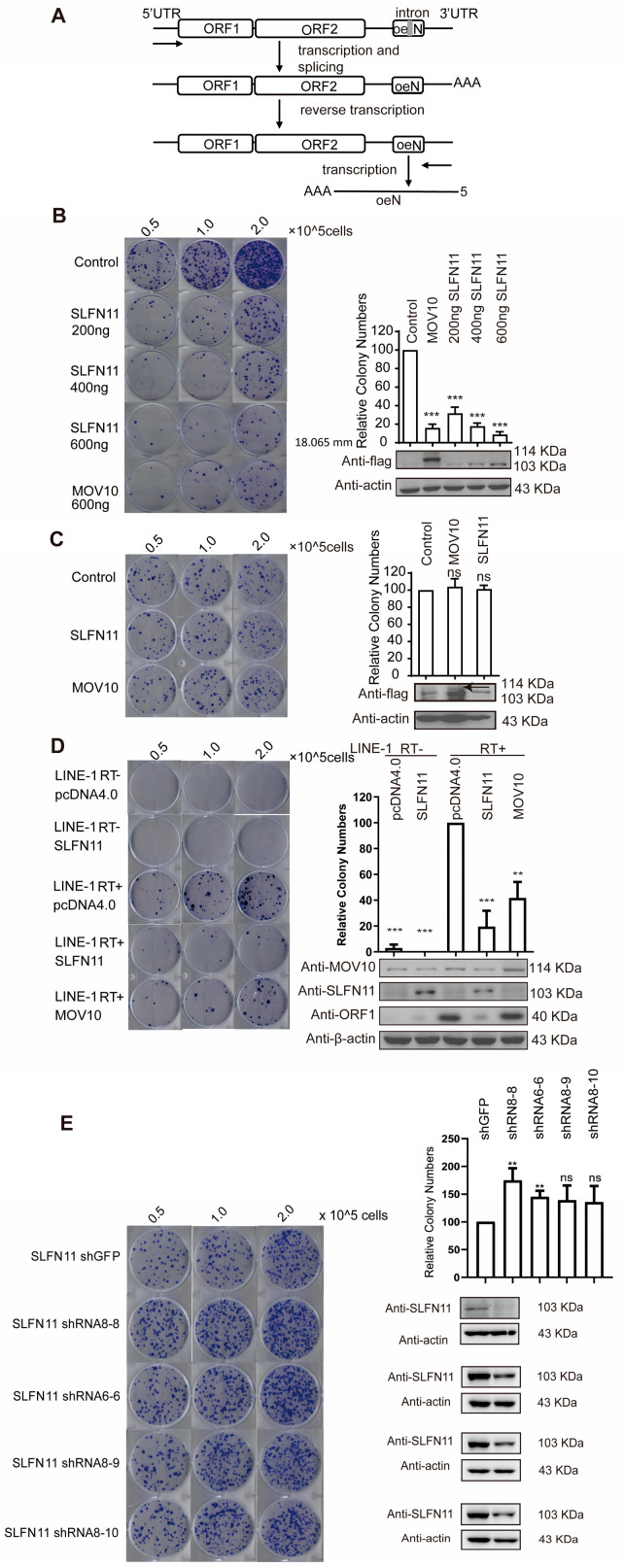
SLFN11 inhibits LINE-1 retrotransposition. (**A**) An illustration of the CMV-L1-neo^RT+^ reporter plasmid and its transcriptional product conferring G418 resistance. (**B**) The ectopic expression of SLFN11 suppresses LINE-1 retrotransposition. HeLa cells cotransfected with CMV-L1-Neo^RT+^ and increasing amounts of the SLFN11 expression plasmid (0, 0.5, 1.0 μg) were subjected to G418 selection. (**Left**) Representative crystal violet-stained colonies. (**Right**) Western blot confirming SLFN11 overexpression (lower panel) and the quantitative analysis of relative colony formation (upper panel, normalized to vector control). MOV10 serves as a loading control. Data represent the relative colony numbers normalized to the SLFN11-negative control (set as 100%). (**C**) SLFN11 expression does not affect basal colony formation efficiency. HeLa cells were transfected with empty pcDNA3.1 and underwent identical G418 selection. Western blot confirmation and colony quantification were performed as described in (**B**). The black arrow indicates the band for MOV10 protein and the same below. (**D**) SLFN11 specifically inhibits RT-competent LINE-1 elements. Hela cells were co-transfected with either CMV-L1-neo^RT−^ or CMV-L1-neo^RT+^ constructs alongside an empty vector, pcDNA4.0 control, or SLFN11 expression plasmid, followed by retrotransposition analysis. (**E**) Endogeggest that SLFN11 may reduce LINnous SLFN11 depletion enhances LINE-1 retrotransposition. SLFN11-knockdown HeLa cells were transfected with CMV-L1-neo^RT+^ and subjected to G418 selection. Crystal violet-stained colonies (representative image) and quantitative data (graph) showed increased retrotransposition upon SLFN11 knockdown. All data were representative of at least three independent experiments. The values are expressed as means ± SDs. Statistical analyses were performed with two-tailed, unpaired Student’s *t*-test (**, *p* < 0.01; ***, *p* < 0.001; and ns, not significant).

To evaluate LINE-1 activity, we utilized the well-characterized LINE-1 retrotransposition assay (Figure 1A). Briefly, we transfected the CMV-L1-neo^RT+^ reporter plasmid into cells, which contains a neomycin resistance gene inserted into the 3′UTR of LINE-1. The neomycin resistance gene is disrupted by an intron oriented in the same transcription direction as LINE-1. This design ensures that the resistance gene can only be expressed after LINE-1 retrotransposition, in which the intron is removed during RNA splicing. Consequently, the number of G418-resistant cell colonies reflects LINE-1 retrotransposition events. The overexpression of SLFN11 markedly reduced the number of G418-resistant colonies, indicating that ectopically expressed SLFN11 significantly inhibits LINE-1 retrotransposition to a similar level as MOV10 [43], which is a well-documented LINE-1 repressor [20,33,46] (Figure 1B). SLFN11 suppressed LINE-1 mobility in a dose-dependent manner (Figure 1B). To rule out the possibility that SLFN11 may inhibit the expression of neomycin resistance gene, we performed the cell colony assay by transfecting the HeLa cells with pcDNA3.1 (harboring an intron-free neomycin resistance gene) and SLFN11, MOV10, or the control vector pcDNA4.0. SLFN11 did not impair G418-resistant colony formations under these conditions (Figure 1C), confirming its specific suppression of LINE-1 activity rather than general interference with neomycin resistance gene expression.

Considering the essential role of reverse transcriptase (RT) activity in the LINE-1 retrotransposition cycle, we engineered an RT-inactive CMV-L1-neo^RT−^ construct containing a D702Y missense mutation within the reverse transcriptase domain of the ORF2 protein [47]. SLFN11 failed to significantly reduce G418-resistant cell colonies in cells transfected with CMV-L1-neo^RT−^ (Figure 1D), suggesting that it serves as a good negative control. To test whether endogenous SLFN11 also represses LINE-1, we performed the shRNA-mediated knockdown of SLFN11. The depletion of SLFN11 resulted in a two-fold increase in G418-resistant colonies (Figure 1E), demonstrating that endogenous SLFN11 constitutively restricts LINE-1 mobility. Collectively, these findings establish SLFN11 as a novel regulator of LINE-1 retrotransposition.

### 3.2. SLFN11 Inhibits Retrotransposition of LTR Retrotransposon IAP and MusD

**Figure 2:**
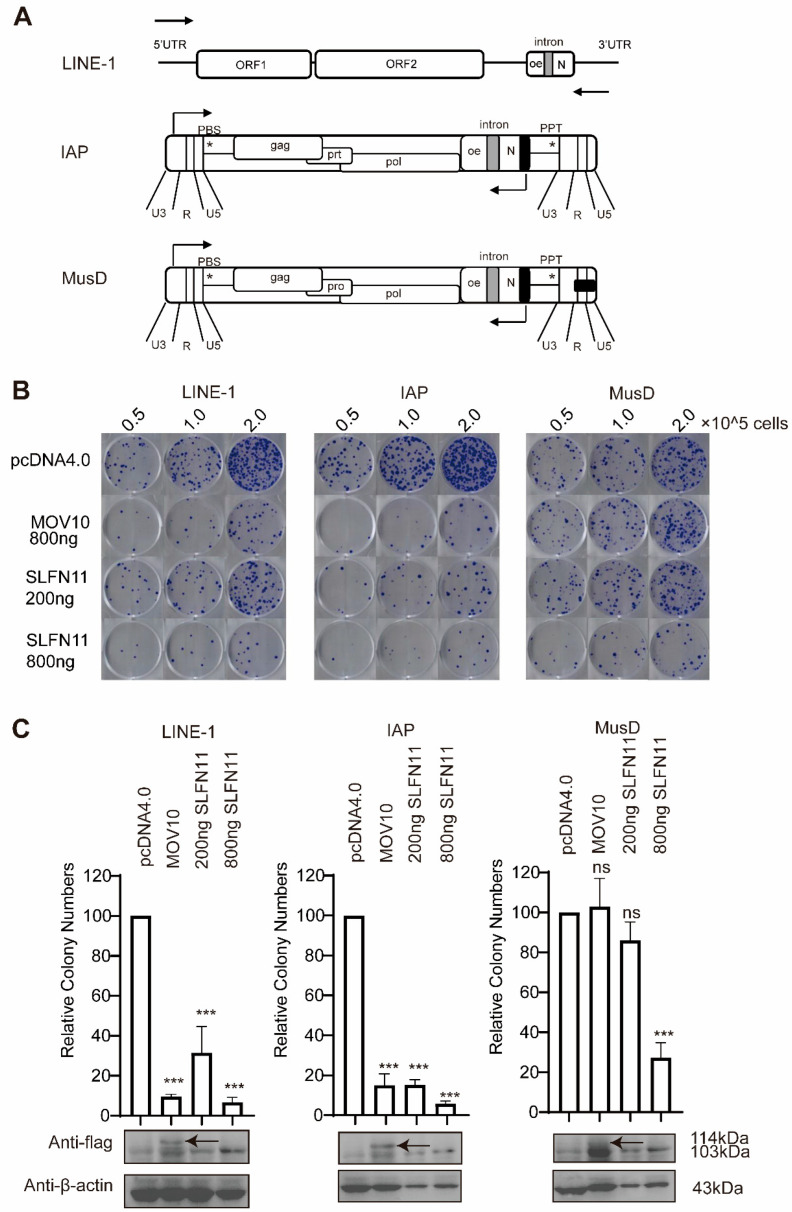
SLFN11 inhibits IAP and MusD retrotransposition. (**A**) Schematic diagrams of IAP-neo^TNF^ and MusD-neo^TNF^ reporter constructs. (**B**,**C**) The ectopic expression of SLFN11 inhibits IAP and MusD retrotransposition. HeLa cells were co-transfected with CMV-L1-neo^RT+^, IAP-neo^TNF^, and MusD-neo^TNF^ along with either 200 ng or 800 ng of SLFN11-Flag, MOV10-Flag (positive control), or equivalent empty vectors of DNA. (**B**) Retrotransposition efficiency was quantified by the G418-resistant colony formation (crystal violet staining). (**C**) Upper panel: the quantitative analysis of retrotransposition events normalized to vector control (set as 100%). Lower panel: Western blot analysis confirming SLFN11 and MOV10 expression levels. Data are representative of at least three independent experiments. The values are expressed as means ± SDs. Statistical analyses were performed with two-tailed, unpaired Student’s *t*-test (*, *p* < 0.05; ***, *p* < 0.001; and ns, not significant).

Most retroelements have lost their mobility due to the accumulation of inactivating mutations. Nonetheless, a subset remains functionally active, including LTR (long-term repeat), murine MusD, and IAP (interacisternal A particles). To determine whether SLFN11 broadly restricts retrotransposons beyond non-LTR elements, we further evaluated the inhibitory activity of SLFN11 against MusD and IAP using two constructs, MusD-neo^TNF^ and IAP-neoTM, both of which carry the neomycin resistance selection marker similarly to CMV-L1-neo^RT+^ (Figure 2A); thus, G418-resistant cell colonies can be generated once retrotransposition occurs [48]. The results showed that the positive control MOV10 can only inhibit LINE-1 and IAP, but not MusD retrotransposition (Figure 2B,C), which is consistent with the previous report [43]. Furthermore, the ectopic expression of SLFN11 can also diminish the number of G418-resistant cell colonies upon transfection with IAP and MusD to different extents, indicating that SLFN11 may also have an inhibitory activity against LTR retrotransposons. However, unlike its effect on LINE-1 and IAP, the ectopic expression of SLFN11 only impacted MusD at high doses. (Figure 2B,C). These results indicated that SLFN11 is a multifunctional retrotransposon restriction factor against both LTR and non-LTR elements.

### 3.3. SLFN11 Diminishes LINE-1 RNA

**Figure 3:**
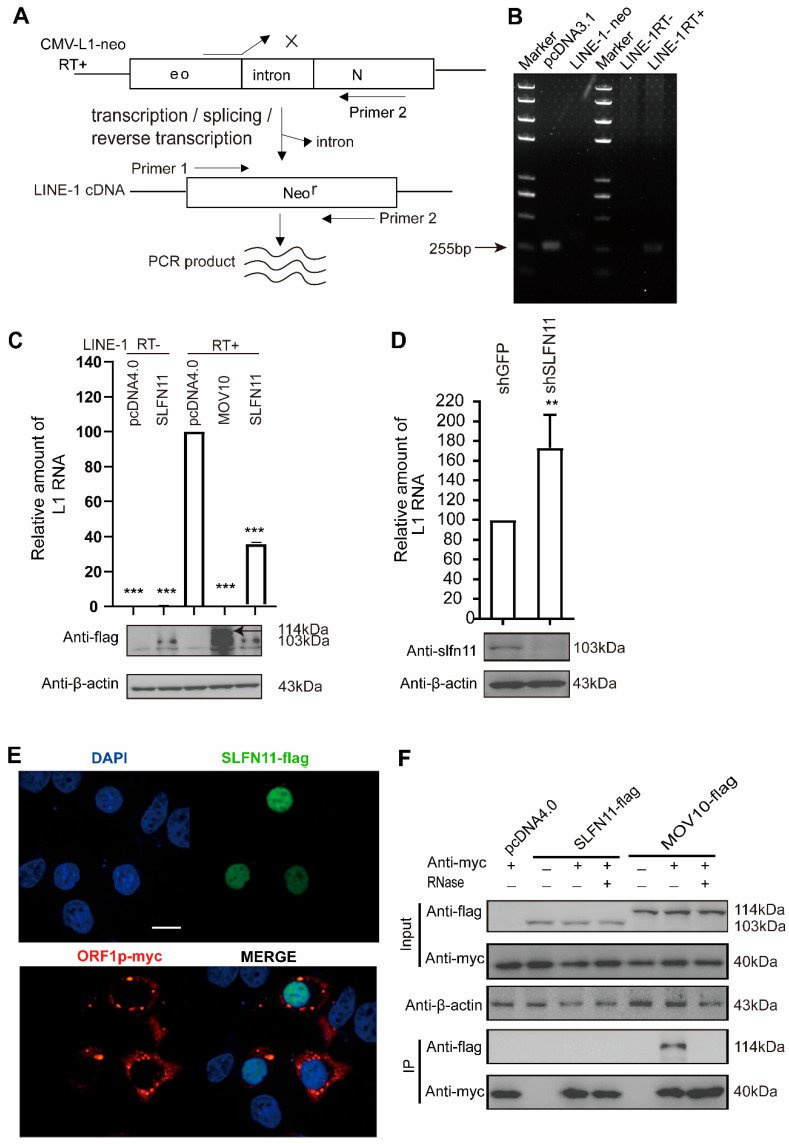
SLFN11 diminishes LINE-1 RNA. (**A**) A schematic of intron-spanning primer design for specific LINE-1 RNA detection. Primers (arrows) that span the Neo cassette intron in the LINE-1 in DNA cannot amplify LINE-1 DNA, as indicated by the cross symbol. Only spliced LINE-1 RNA can be amplified to eliminate potential DNA contamination from the transfected plasmid. (**B**) The intron-containing CMV-L1-Neo^RT+^ plasmid cannot be amplified by PCR using intron-spanning primers targeting the neomycin resistance sequence but successfully amplifies spliced L1 RNA products from successful retrotransposition events. (**C**) Ectopic SLFN11 expression decreases LINE-1 mRNA. HeLa cells were co-transfected with SLFN11 and CMV-L1-Neo^±^ constructs. LINE-1 RNA levels analyzed by RT-qPCR (72 h post-transfection). (**D**) Endogenous SLFN11 inhibits LINE-1 mRNA. The figure shows 293FT cells with shSLFN11 knockdown transfected with CMV-L1-neo^RT+^ and LINE-1 RNA quantified by RT-qPCR (72 h). (**E**) Subcellular localization analysis of SLFN11 and ORF1p. HeLa cells expressing Flag-SLFN11 and Myc-ORF1p immunostained with anti-Flag (green) and anti-Myc (red). Scale bars, 10 μm. (**F**) SLFN11 does not interact with ORF1p. Myc-ORF1p co-expressed with Flag-SLFN11 or Flag-MOV10 in 293T cells. RNase-treated lysates immunoprecipitated with anti-Myc; co-precipitated proteins analyzed by immunoblotting. Data are representative of at least three independent experiments. The values are expressed as means ± SDs. Statistical analyses were performed with two-tailed, unpaired Student’s *t*-test (**, *p* < 0.01; ***, *p* < 0.001).

LINE-1 RNA plays a central role during its retrotransposition, serving both as mRNA for protein expression and as the template to produce new cDNA copies. LINE-1 RNA levels directly regulate the expression of ORF1p and ORF2p and determine the abundance of LINE-1 cDNA. In order to avoid the influence of endogenous LINE-1 on the experimental results, we employed a pair of primers that solely targeted spliced LINE-1 RNA [21]. As shown in Figure 3A,B, the forward primer was designed to anneal exclusively to the exon–exon junction of the spliced neomycin resistance gene, ensuring the amplification of spliced LINE-1 RNA only, as previously reported [45]. To confirm primer specificity, we tested two constructs: CMV-L1-neo^RT+^ (containing an intron within the neomycin resistance gene) and pcDNA3.1 (lacking the intron). The PCR analysis shows that these primers amplified the intron-free neomycin resistance gene only in pcDNA3.1 (Figure 3B left), which is consistent with the inability to amplify CMV-L1-neo^RT+^ due to its retained intron. This specificity was further validated by RT-PCR targeting spliced RNA (Figure 3B right). We next examined the effect of SLFN11 on LINE-1 RNA using RT-qPCR. The ectopic expression of SLFN11 decreased LINE-1 mRNA by ~three-fold, while the positive control MOV10 almost diminished LINE-1 mRNA, similar to previous reports [49] (Figure 3C). Conversely, when we depleted endogenous SLFN11 in cells with shRNA oligonucleotides, LINE-1 mRNA increased ~two-fold (Figure 3D), indicating that both exogenous and endogenous SLFN11 can decrease the LINE-1 RNA level.

These results suggest that SLFN11 may reduce LINE-1 RNA levels by either suppressing transcription or promoting RNA degradation. However, the data of immunofluorescence staining showed that SLFN11 is mainly located in the nucleus, consistent with its reported nuclear functions [31,50], whereas LINE-1 ORF1p is dispersed in the cytoplasm (Figure 3E), suggesting that SLFN11 is unlikely to affect the stability of LINE-1 RNA through interactions with LINE-1 RNP in the cytoplasm, in contrast to MOV10, which binds ORF1p in an RNA-dependent manner (Figure 3F) [20]. Collectively, these data support a nuclear mechanism for SLFN11-mediated LINE-1 suppression.

### 3.4. SLFN11 Represses the Transcription of LINE-1

**Figure 4:**
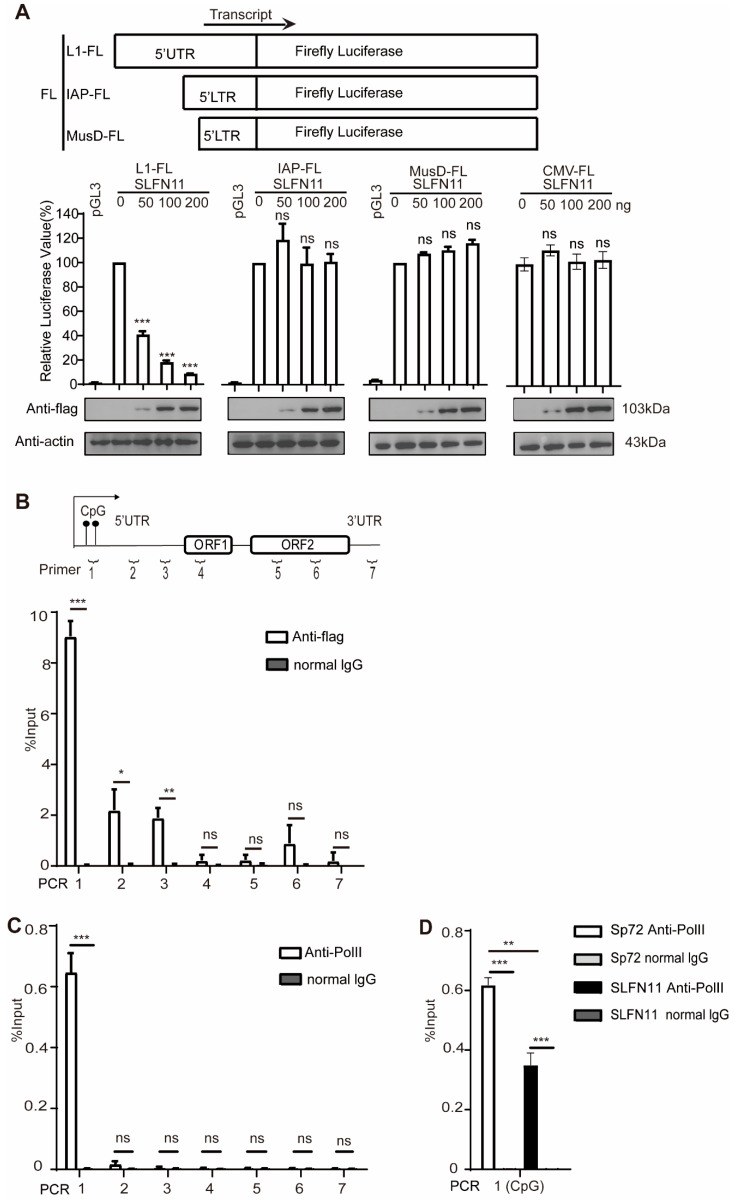
SLFN11 represses the transcription of LINE-1. (**A**) SLFN11 specifically suppresses the LINE-1 promoter. The 293T cells were co-transfected with SLFN11 DNA and luciferase reporters driven by LINE1-FL, IAP-FL, or MusD-FL promoters (schematic, top). Firefly luciferase activity reflects LINE-1 5′UTR promoter activity, with the promoterless pGL3-basic vector as the negative control. Firefly luciferase activity reflects 5′UTR promoter activity, with the promoterless pGL3-basic vector as the negative control. SLFN11 dose-dependently inhibited the LINE-1 promoter (left) but showed no significant effect on IAP (middle), MusD (right), or CMV-driven luciferase expression. SLFN11 protein levels were verified by Western blot (bottom). Baseline luciferase activity (no SLFN11) was defined as 100%. (**B**) SLFN11 is associated with endogenous LINE-1 5′UTR regions. The schematic shows primer locations (top) for amplifying genomic LINE-1 elements. DNA co-immunoprecipitated with FLAG-tagged SLFN11 from MCF7 cells was quantified using seven primer pairs spanning LINE-1 loci. (**C**,**D**) SLFN11 impairs RNA polymerase II (Ser5-P) recruitment to LINE-1 5′UTR loci. (**C**) The ChIP analysis of RNA polymerase II (Ser5-P) binding regions on LINE-1 DNA. ChIP assays were conducted in MCF7 cells to investigate RNA polymerase II binding at LINE-1 elements, employing specific antibodies against Ser5-phosphorylated RNA polymerase II (Ser5-P) and control IgG, along with primers targeting defined regions of the LINE-1 locus. (**D**) SLFN11 impairs the binding of RNA polymerase II (Ser5-P) to LINE-1 retrotransposon DNA. MCF7 cells were transfected with either SLFN11 or the empty pSP72 vector. ChIP was performed using antibodies against RNA polymerase II (Ser5-P) or normal IgG along with primers targeting defined regions of the LINE-1 locus. Data are representative of at least three independent experiments. The values are expressed as means ± SDs. Statistical analyses were performed with two-tailed, unpaired Student’s *t*-test (*, *p* < 0.05; **, *p* < 0.01; ***, *p* < 0.001; and ns, not significant).

Given the nuclear localization of SLFN11 and its lack of association with LINE-1 ribonucleoprotein (RNP) complexes, we hypothesized that SLFN11 might regulate LINE-1 transcription in the nucleus. Since LINE-1 transcription is initiated by its intrinsic promoter situated within the 5′-UTR, we examined whether SLFN11 modulates the activity of the LINE-1 internal promoter. We generated a series of luciferase reporter constructs by inserting the 5′-UTR sequences of LINE-1, IAP, and MusD retrotransposons or the CMV promoter upstream of the firefly luciferase gene in pGL3 plasmids, as previously described [20]. Strikingly, ectopically expressed SLFN11 strongly decreased the luciferase activity driven by LINE-1 5′-UTR (Figure 4A), demonstrating that SLFN11 efficiently suppresses LINE-1 promoter activity [20]. In contrast, little effect of SLFN11 was observed on the CMV promoter and LTR promoter activities of IAP and MusD retrotransposons (Figure 4A), suggesting distinct regulatory mechanisms for LINE-1 versus LTR-driven retrotransposons. To elucidate the molecular mechanism underlying SLFN11-mediated LINE-1 repression, we next performed the chromatin immunoprecipitation (ChIP) assay in MCF7 cells using seven primer pairs spanning the LINE-1 element (Figure 4B). The MCF7 breast cancer cell line endogenously expresses relatively lower levels of SLFN11 and abundant full-length LINE-1 transcripts [51,52], making them suitable for ChIP analysis. Furthermore, this cell line exhibits elevated ORF2 mRNA levels and reverse transcriptase activity compared to most other cell lines [51], indicating the robust transcriptional and functional activity of LINE-1 elements.

ChIP assays revealed the specific enrichment of SLFN11 at the 5′-UTR of LINE-1 loci (Figure 4B), confirming its previously characterized DNA-binding activity [30]. This binding pattern might overlap with the spatial distribution of serine 5-phosphorylated RNA polymerase II (RNA Pol II Ser5-P) at LINE-1 promoters (Figure 4C), which is crucial for the initiation of LINE-1 transcription. To determine whether SLFN11 binding interferes with RNA Pol II recruitment, we performed additional ChIP experiments. As expected, SLFN11 was found to decrease the recruitment of RNA polymerase II(Ser5-P) to the LINE-1 5′UTR promoter (Figure 4D), suggesting that SLFN11 attenuates LINE-1 transcription by disturbing the association between the LINE-1 5′UTR promoter and RNA polymerase II.

### 3.5. SLFN11 Promotes the Formation of Compact Heterochromatin

**Figure 5:**
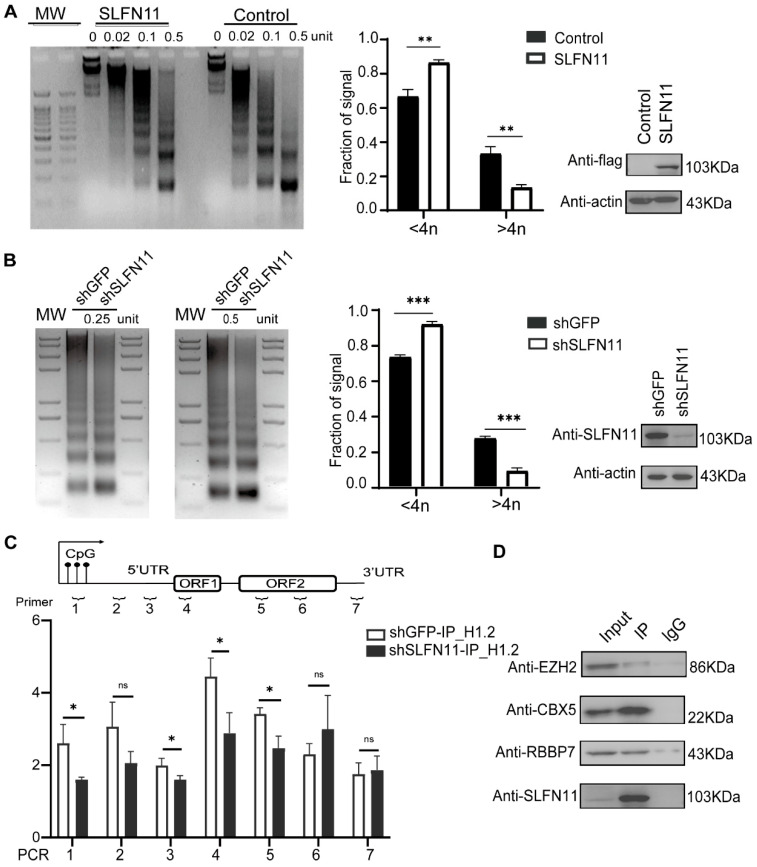
SLFN11 promotes higher-order heterochromatin assembly. (**A**) Nucleosome organization in SLFN11-overexpressing MCF7 cells. MCF7 cells transfected with pcDNA4.0-SLFN11 or an empty vector were treated with increasing MNase concentrations (0, 0.02, 0.1, 0.5 U). MNase-digested DNA was resolved by agarose gel electrophoresis. DNA fragments corresponding to smaller tetranucleosomes (≤4n) or larger pentanucleosomes (>4n) at 0.5 U MNase were quantified using ImageJ (Version 1.53t, NIH, Bethesda, MD, USA) and normalized to the total lane signal. (**B**) Nucleosome stability in SLFN11-deficient 293FT cells. Wild-type or SLFN11-knockdown (KD) 293FT cells were treated with MNase (0, 0.02, 0.1, 0.5 U). Fragment size distribution at 0.5 U MNase was analyzed as shown in (**A**). (**C**) SLFN11 promotes the binding of the linker histone H1.2 in LINE-1 loci. H1.2 occupancy at LINE-1 regions was assessed by ChIP-qPCR in wild-type versus SLFN11-KD 293FT cells using anti-H1.2 antibodies. (**D**) SLFN11 interacts with heterochromatin-associated factors. Co-IP assays were used in 293FT cells using anti-SLFN11 antibodies followed by immunoblotting for indicated proteins. Data represent the mean ± SD from ≥3 independent experiments. Statistical significance was determined by two-tailed unpaired Student’s *t*-test (* *p* < 0.05, ** *p* < 0.01, *** *p* < 0.001; ns, not significant).

SLFN11 has been reported to act as a global regulator of chromatin structure and an intrinsic immediate-early gene (IEG) activator in response to replicative stress [32]. Given that SLFN11 inhibits LINE-1 transcription, it is tempting to ask whether SLFN11 influences epigenetic modification, such as the formation of heterochromatin, to affect LINE-1 transcription, consequently inhibiting LINE-1 retrotransposition. We analyzed chromatin compaction using micrococcal nuclease (MNase) digestion. MNase preferentially cleaves linker DNA between nucleosomes, generating mononucleosomal fragments; tightly packed chromatin resists MNase digestion. MNase digestion assays in MCF7 cells revealed the formation of higher-order chromatin architecture in SLFN11-overexpressing cells (Figure 5A). Notably, genomic DNA from SLFN11-knockdown (KD) 293FT cells exhibited significantly increased MNase sensitivity compared to wild-type (WT) controls (Figure 5B), suggesting that the depletion of SLFN11 contributed to increased chromatin accessibility compared with WT 293FT cells. This indicates that SLFN11 plays an important role in promoting the formation of genome-wide heterochromatin.

To further examine whether SLFN11 can directly impact LINE-1 elements on chromatin, we re-analyzed the available ATAC-seq (Assay for Transposase-Accessible Chromatin with high throughput sequencing) data [32] obtained from human leukemia CCRF-CEM SLFN11-positive cells and the corresponding SLFN11-KO cells. The result shows that LINE-1 repeats in chromatin are more accessible in SLFN11-KO cells compared to leukemia CCRF-CEM SLFN11-positive cells (Figure S1), reflecting the H3K9me3-dependent chromatin tightening required for drug-tolerant cancer cell survival [53]. Taken together, we concluded that SLFN11 has a direct effect on the formation of repressed LINE-1 heterochromatin.

Moreover, we found that SLFN11 can directly interact with NPM1 (Table S1), which restricts LINE-1 via the deposition of histone H1 and hinders the initiation of transcription, which is similar to the physiological function of the Dot1l-NPM1-H1 complex [54]. The knockdown of SLFN11 had a broad impact on the binding of linker histone H1.2 to the LINE-1 sequence, suggesting that SLFN11 promotes H1.2 binding on LINE-1 loci (Figure 5C). Furthermore, SLFN11 associated with heterochromatin, organizers HP1α (CBX5) and Polycomb Repressor Complex 2 (PRC2) components EZH2/RBBP7 (Figure 5D), implicating dual H3K9me3- and H3K27me3-mediated repression. Immunoprecipitation-coupled mass spectrometry (IP-MS) results also confirmed specific interactions with NPM1, H1.2, and RBBP7 (Table S1). Collectively, these data suggest that SLFN11 promotes heterochromatin formation, thereby reducing the occupancy of RNA PolII in the promoter of LINE-1.

### 3.6. The Helicase Domain Is Indispensable for the SLFN11-Mediated Repression of LINE-1

**Figure 6:**
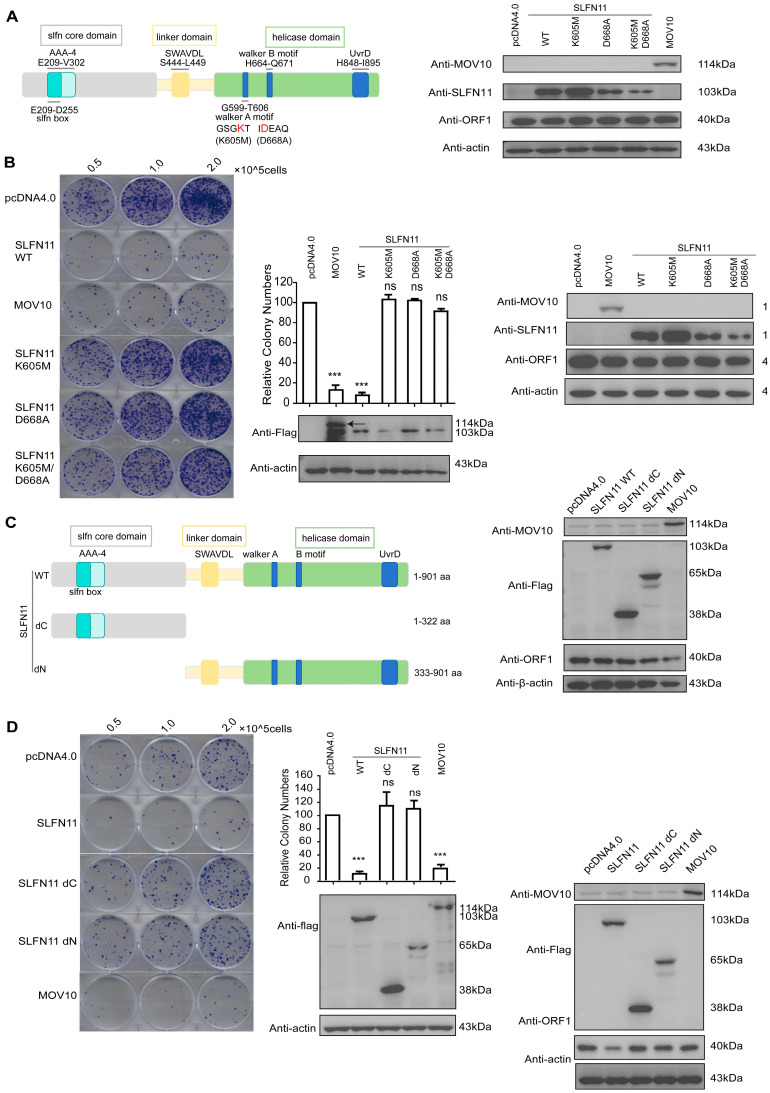
The helicase domain of SLFN11 is required for the suppression of LINE-1 retrotransposition. (**A**) A schematic of the SLFN11 helicase structure. SLFN11 contains conserved motifs, including the SLFN box, AAA-4 domain, SWAVDL motif, Walker A/B motifs, and a UvrD-like helicase domain. Critical residues in Walker A (GSGKT; K605M) and Walker B (IDEAQ; D668A) are highlighted in red. Neither single (K605M, D668A) nor double (K605M/D668A) mutations significantly affect endogenous ORF1p levels. (**B**) Helicase-inactive SLFN11 mutants fail to suppress LINE-1 activity. Left: A representative colony formation assay following transfection with wild-type (WT) or mutant SLFN11. Right: The quantification of neomycin-resistant colonies normalized to empty vector control (set as 100%). Western blot confirms comparable SLFN11 mutant expression and unchanged ORF1p levels. (**C**) The schematic illustration of SLFN11 truncation constructs. Neither N-terminal (ΔN) nor C-terminal (ΔC) truncations alter endogenous ORF1p expression. (**D**) Truncation constructs of SLFN11 lack retrotransposition inhibitory activity. The colony formation assay with CMV-L1-neo^RT+^ in the presence of SLFN11 truncations is shown in the left panel. Data represent the mean ± SD from ≥3 independent experiments. Statistical significance was determined by two-tailed unpaired *t*-test (*** *p* < 0.001; ns, not significant).

SLFN11 contains an SLFN core domain in N-termini, which is a linker domain with the SWAVDL motif and the C-terminal helicase domain (Figure 6A). The C-terminal portion of SLFN11 possesses a conserved putative DNA/RNA helicase, which was reported to exhibit ATP-dependent DNA or RNA structure remodeling activity [50]. Considering that SLFN11 can associate with the LINE-1 internal promoter, it is plausible to think that the helicase structure might perform a certain function in restricting LINE-1 mobility. To test this, we focused on two conserved helicase motifs, including Walker A (Motif I) and Walker B (Motif II), which are critical for ATP binding and hydrolysis across species [50].

Site-directed mutagenesis was performed to disrupt these motifs by substituting Lys605 (Walker A) and Asp668 (Walker B), generating single (K605M, D668A) and double (K605M/D668A) mutants (Figure 6A). Consistent with prior findings, these mutations abrogate SLFN11′s ATPase activity [50]. Notably, none of the mutants significantly altered endogenous ORF1p protein levels (Figure 6B). Retrotransposition assay data showed that these mutants failed to inhibit LINE-1 retrotransposition or reduce ORF1p expression (Figure 6B), demonstrating that the helicase-like structure of SLFN11 is important for restricting LINE-1. These results are consistent with previous studies demonstrating that Asp668 and Glu669 bind to Mg^2^^+^ and facilitate ATP hydrolysis; these are mechanisms that are essential for retroviral restriction [38].

This is consistent with structural studies [38] demonstrating that Asp668 and Glu669 bind to Mg^2^^+^ and facilitate ATP hydrolysis, which are mechanisms that are essential for retroviral restriction. Immunofluorescence (IF) analysis showed the cytoplasmic mislocalization of the dN mutant (Figure S2), suggesting that nuclear localization—dependent on N-terminal sequences—is critical for LINE-1 repression.

## 4. Discussion

Although retrotransposons make up roughly 40% of the mammalian genome, they are predominantly confined to highly condensed heterochromatin [55]. The transcription of these repetitive genomic sequences is tightly repressed during embryonic development [56]. The establishment and maintenance of the heterochromatin environment represent a key epigenetic strategy for silencing these repetitive elements. In this study, we demonstrate that a human Schlafen family protein, SLFN11, acts as a modifier that specifically restricts the transcription of endogenous retrotransposable LINE-1s.

Interestingly, we also found out that SLFN11 inhibited IAP and MusD elements without suppressing their transcription, suggesting distinct mechanisms of action compared to its repression of LINE-1. Notably, IAP and MusD belong to endogenous retroviruses, which differ from non-LTR-like LINE-1 in both their genome architecture and replication mechanisms. This functional dichotomy is consistent with previous findings that SLFN11 inhibits HIV-1 replication through the selective translational repression of viral proteins rather than transcriptional silencing [57]. Furthermore, SLFN11 has been shown to reduce protein synthesis from exogenous reporters (e.g., GFP) and endogenous genes (e.g., Vinculin and GAPDH) in uninfected cells [57]. These findings raise critical questions regarding whether the SLFN11-mediated inhibition of IAP/MusD involves translational control or alternative pathways, which is a subject warranting further investigation.

SLFN11 is a putative DNA/RNA helicase implicated in chromatin remodeling and IEG activation in response to DNA damage [32]. Our data indicate that the helicase domain is also necessary for SLFN11 to inhibit LINE-1 retrotransposition. While the SLFN11 dN mutant (333–901 aa) retains the helicase domain, it fails to suppress LINE-1 activity. This deficiency correlates with its inability to locate in the nucleus, which is consistent with one of our unpublished results indicating that the NLS is located in the N-terminal domain of SLFN11. More importantly, structural studies indicate that SLFN11 recognizes and binds to DNA/RNA via its N-terminal SLFN core domain (1–354 aa) [30]. Thus, the dN mutant likely lacks both nuclear targeting and nucleic acid recognition capacities, which are two prerequisites for LINE-1 inhibition.

This interpretation aligns with reports that both N-terminal ribonuclease/ATPase activity and C-terminal helicase activity are required for SLFN11-mediated retroviral inhibition (e.g., PFV) and ribosome biogenesis impairment [36,38].

However, it is still not clear how SLFN11 promotes the formation of condensed heterochromatin. Histone tail modifications are well-recognized mechanisms of heterochromatinization, which has also been identified by several research groups to repress LINE-1s [12]. Specifically, the heterochromatin protein 1(HP1) directly binds to H3K9me3 and is responsible for the establishment, propagation, and maintenance of higher-order-condensed chromatin structures [58,59]. Our findings reveal a novel interaction between SLFN11 and heterochromatin protein 1α (HP1α/CBX5) (Figure 5D), indicating that SLFN11 may be involved in H3K9me3-mediated LINE-1 repression (Figure 5D).

Furthermore, the linker histone H1.2, recognized for its cooperative role with histone methyltransferases (SETDB1, Suv39h1, and Suv39h2) in facilitating H3K9me3 deposition [60], additionally interacts with H3K27me3-marked nucleosomes to promote chromatin compaction [61,62,63]. Our data demonstrate that SLFN11 facilitates the binding of H1.2 to the LINE-1 promoter (Figure 5C) and promotes the interaction of H1.2 with PRC2 components (EZH2 and RBBP7) (Figure 5D). As PRC2 catalyzes H3K27 trimethylation, these observations collectively suggest that SLFN11 may orchestrate heterochromatin formation through the dual engagement of H3K9me3 and H3K27me3 pathways, potentially via H1.2-mediated chromatin compaction. This proposed mechanism warrants further investigation to delineate the hierarchical relationships between these epigenetic modifications.

## 5. Conclusions

In conclusion, our study shows that SLFN11 suppresses LINE-1 retrotransposition through epigenetic silencing. Mechanistically, SLFN11 promotes the formation of higher-order heterochromatin assembly and prevents the recruitment of RNA polymerase II to the LINE-1 promoter, thereby suppressing LINE-1 transcription. Domain characterization revealed that helicase domains are indispensable for the SLFN11-mediated repression of LINE-1. These findings not only advance our understanding of host defense mechanisms against LINE-1 but also highlight SLFN11 as a potential therapeutic target for LINE-1-associated pathologies.

## Data Availability

All data generated or analyzed during this study are included in this article (and its Supplementary Information Files).

## Supplementary Materials

The following supporting information can be downloaded at https://www.mdpi.com/article/10.3390/cells14110790/s1, Figure S1: SLFN11 promote the repressed chromatin over LINE-1 repeats; Figure S2: The location of SLFN11 truncation; Table S1: Interactions of SLFN11 with NPM1, H1.2, and RBBP7 Identified by Mass Spectrometry.

## Abbreviations

The following abbreviations are used in this manuscript:

LINE-I	Long interspersed nuclear element-I
EN	Endonuclease
RT	Reverse transcriptase
UTR	Untranslated regions
ATP	Adenosine triphosphate
HUSH	Human-silencing hub complex
KAP1	KRAB-associated protein 1
MOV10	Moloney leukemia virus type 10 protein
ZAP	Zinc-finger antiviral protein
ISG	Interferon-stimulated gene
FBS	Fetal bovine serum
LTR	Long terminal repeat
ORF	Open reading frame
RNP	Ribonucleoprotein complexes
PCR	Polymerase Chain Reaction
qPCR	Quantitative Real-Time PCR
PVDF	Polyvinylidene Fluoride
rpm	Revolutions per minute
SD	Standard deviation
SDS-PAGE	SDS-Polyacrylamide gel electrophoresis
Tris	Trihydroxymethylaminomethane
PFV	Prototype foamy virus
EIAV	Equine infectious anemia virus
HIV-1	Human immunodeficiency virus 1
WNV	West Nile virus
DENV	Dengue virus
ZIKV	Zika virus
