# Supplementary material for: SLFN11 Restricts LINE-1 Mobility
Source: Cells. 2025 May 28;14(11):790. doi: 10.3390/cells14110790 (PMC12153781; doi:10.3390/cells14110790)
Supplement: Supplementary file 1 [file cells-14-00790-s001.zip › cells-3489798-supplementary.pdf]

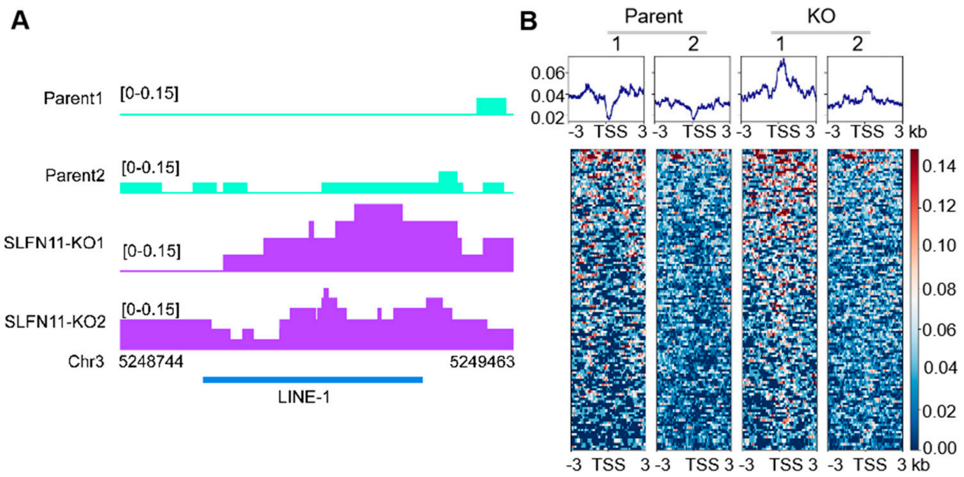

Figure S1. SLFN11 promote the repressed chromatin over LINE-1 repeats. Human leukemia CCRF-CEM SLFN11-positive cells and the SLFN11-KO cells were subjected to ATAC-seq. (A) Representative sequencing tracks of ATAC-seq for the LINE-1 loci at chromatin 3 (Chr3) in CCRF-CEM SLFN11-KO (purple) and parental (light green) cells. (B) Heatmaps showing the ATAC-seq signal intensity at LINE-1 promoter regions (defined as -3kb and +3kb of the transcription start site (TSS)). The signal intensity at TSS in SLFN11-KO cells are higher than 0.04. whereas it is lower than 0.4 in SLFN11 positive cells, suggesting that SLFN11 can promote the formation of repressed chromatin over LINE-1 promoter.

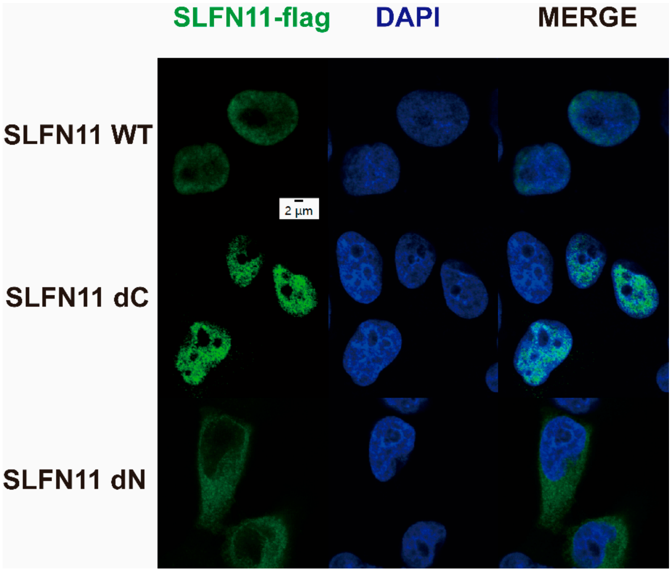

Figure S2. The location of SLFN11 truncation. HeLa cells are transfected with flag-SLFN11-WT, flag-SLFN11-dC and flag-SLFN11-dN, 48 hours post-transfection, cells are stained with rabbit anti-flag (green) followed by FITC-labeled Goat anti-Rabbit antibody used as secondary antibodies. Data are representative of at least three independent experiments.

					161434-LINE-1				161434-SLFN11						
Gene Symbol	other name	UniProt Entry	Accession	Description	Score	Coverage	Peptides	PSM	Score	Coverage	Peptides	PSM	AAs	MW [kDa]	calc. pI
SLFN11		SLN11_HUMAN	157388957	schlafen family member 11 [Homo sapiens]	0.00	0.00			183.34	29.19	22	61	901	102.8	7.77
NPM1		NPM_HUMAN	40353734	nucleophosmin isoform 2 [Homo sapiens]	0.00	5.28	1	1	12.48	15.85	3	5	265	29.4	4.61
H1-2		H12_HUMAN	4885375	histone H1.2 [Homo sapiens]	0.00	0.00			3.50	15.49	4	4	213	21.4	10.93
RBBP7		RBBP7_HUMAN	4506439	histone-binding protein RBBP7 isoform 2 [Homo sapiens]					0.00	4.24	2	2	425	47.8	5.05

Table S1. Interactions of SLFN11 with NPM1, H1.2, and RBBP7 Identified by Mass Spectrometry.
